# CRISPR/Cas9 targeting of passenger single nucleotide variants in haploinsufficient or essential genes expands cancer therapy prospects

**DOI:** 10.1038/s41598-024-58094-8

**Published:** 2024-03-28

**Authors:** Hakhyun Kim, Jang Hee Han, Hyosil Kim, Minjee Kim, Seung-il Jo, NaKyoung Lee, Seungbin Cha, Myung Joon Oh, GaWon Choi, Hyun Seok Kim

**Affiliations:** 1 Department of Biomedical Sciences, Yonsei University College of Medicine, Seoul, 03722 Korea; 2 Graduate School of Medical Science, Brain Korea 21 Project, Yonsei University College of Medicine, Seoul, 03722 Korea; 3 Department of Urology, Seoul National University Hospital, Seoul, 03080 Korea

**Keywords:** Cancer genomics, Targeted therapies

## Abstract

CRISPR/Cas9 technology has effectively targeted cancer-specific oncogenic hotspot mutations or insertion–deletions. However, their limited prevalence in tumors restricts their application. We propose a novel approach targeting passenger single nucleotide variants (SNVs) in haploinsufficient or essential genes to broaden therapeutic options. By disrupting haploinsufficient or essential genes through the cleavage of DNA in the SNV region using CRISPR/Cas9, we achieved the selective elimination of cancer cells without affecting normal cells. We found that, on average, 44.8% of solid cancer patients are eligible for our approach, a substantial increase compared to the 14.4% of patients with CRISPR/Cas9-applicable oncogenic hotspot mutations. Through in vitro and in vivo experiments, we validated our strategy by targeting a passenger mutation in the essential ribosomal gene *RRP9* and haploinsufficient gene *SMG6*. This demonstrates the potential of our strategy to selectively eliminate cancer cells and expand therapeutic opportunities.

## Introduction

Somatic mutations in cancer have been extensively cataloged by numerous studies, including The Cancer Genome Atlas (TCGA) and the International Cancer Genome Consortium^1–4^. These cancer-specific mutations provide unique therapeutic opportunities for targeted DNA cleavage using CRISPR/Cas9 technology. Early studies have primarily focused on targeting oncogenic mutations. For instance, the specific cleavage of either *EGFR* or *KRAS* hotspot mutation resulted in effective tumor regression^5,6^. Additionally, the simultaneous cleavage of the intronic regions of the fusion oncogenes, such as *EWSR-FLI1* and *BCR-ABL*, selectively eliminated cancer cells^7^. Despite these efforts, tumors lacking oncogenic mutations are not amenable to these approaches. More recently, the generation of multiple double-strand breaks through the simultaneous cleavage of multiple cancer-specific insertion–deletions (InDels) has shown selective cancer cell death^8^. However, only a small fraction of patients harbors these mutations. Therefore, expanding the target mutation spectrum is crucial for increasing the eligible patient population for CRISPR cancer therapy.

## Results

### Targeting passenger SNVs in haploinsufficient or essential genes is an attractive strategy for CRISPR/Cas9-mediated cancer gene therapy

**Figure 1:**
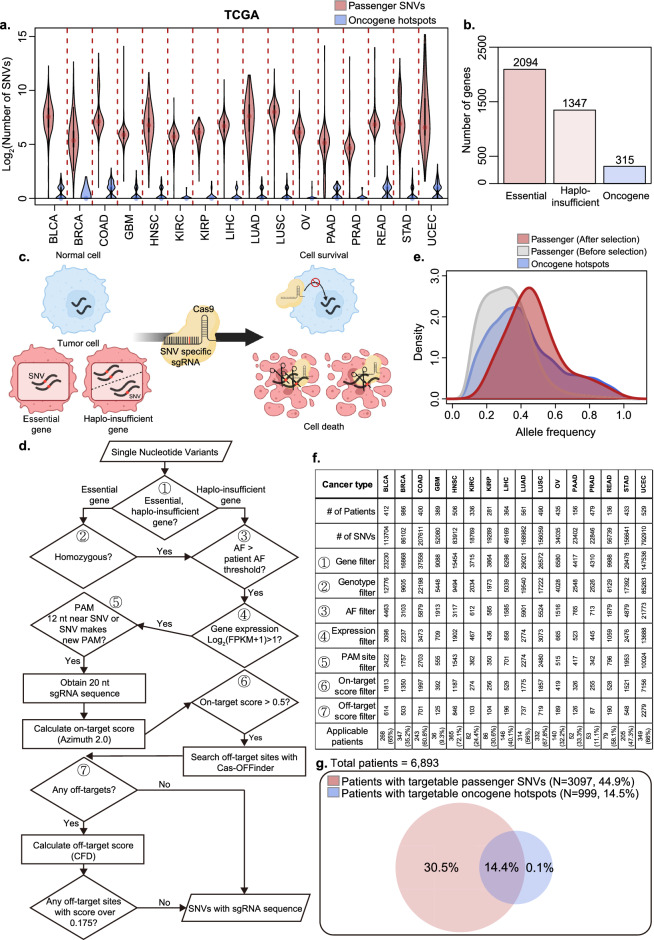
Targeting passenger single-nucleotide variants (SNVs) in haploinsufficient or essential genes is an attractive strategy for CRISPR/Cas9-mediated cancer gene therapy. (**a**) The number of passenger SNVs and oncogene hotspot mutations in each patient in The Cancer Genome Atlas (TCGA) dataset by 16 cancer types (BLCA, BRCA, COAD, GBM, HNSC, KIRC, KIRP, LIHC, LUAD, LUSC, OV, PAAD, PRAD, READ, STAD, UCEC). Violin plots represent the density distributions. (**b**) The number of essential and haploinsufficient genes and oncogenes in human genome. (**c**) Schematic of CRISPR/Cas9-mediated cancer gene therapy by targeting tumor cell-specific passenger SNVs in haploinsufficient or essential genes. (**d**) Flowchart depicting the generation of actionable single-guide RNAs (sgRNAs) targeting passenger SNVs in haploinsufficient or essential genes. (**e**) Density plot comparing the allele frequencies of selected passenger SNVs in haploinsufficient or essential genes (red) and oncogene hotspot mutations (blue). The allele frequency of the entire set of SNVs in haploinsufficient or essential genes before selection is shown in grey. (**f**) The number of selected passenger SNVs in 16 TCGA cancer types after each step of d and the final number of applicable patients. (**g**) Venn diagram illustrating the percentage of eligible patients using this method compared to oncogene hotspot mutations among 16 TCGA cancer types. An applicable patient was defined as one with at least one available qualified sgRNA.

By analyzing the mutation data of each patient in the TCGA dataset, we found that there were significantly more passenger single nucleotide variants (SNVs) than previously reported oncogene hotspot mutations^9^ (Fig. 1a). In this study, a gene is defined as essential when the complete loss of its function compromises viability, or as haploinsufficient when the loss of a single copy is sufficient to compromise viability. Considering that there are approximately 3000 haploinsufficient or essential genes in the human genome, which is ten times more than the oncogenes^10–13^ (Fig. 1b, Supplementary Data 1), passenger SNVs in these genes present a plausible therapeutic target for highly specific CRISPR/Cas9 technology. This strategy could selectively eliminate cancer cells without affecting normal cells that do not harbor these mutations (Fig. 1c). To achieve this, we developed a streamlined data analysis workflow to identify eligible SNVs from a list of mutations in patients (Fig. 1d; see Methods for details). We retained homozygous SNVs for essential genes because only homozygous knockouts would eliminate their functions (Fig. 1d, Steps 1 and 2). On the other hand, for haploinsufficient genes, we kept heterozygous SNVs as well since heterozygous knockout is enough to disrupt normal function (Fig. 1d, Step 1). To select passenger mutations that arose in the early stages of tumor evolution, we selected SNVs that surpassed the patient-tailored allele frequency (AF) threshold, calculated by summing the median and median absolute deviation (MAD) of AF across all heterozygous SNVs in the patient’s data (Fig. 1d, Step 3). Further selection based on gene expression was performed to retain the genes expressed at sufficient levels (Fig. 1d, Step 4). Next, we identified SNVs predicted to be highly efficient CRISPR/Cas9 targets with minimal risk of unintended off-target cleavage (Fig. 1d, Steps 5–7). We first selected SNVs that either created a new protospacer adjacent motif (PAM, NGG where N is any nucleotide) sequence or were within 12 base pairs of an existing PAM site (Fig. 1d, Step 5), as prior research has demonstrated that these sequences determine Cas9 recognition specificity^14^. The adjacent 20-nucleotide single-guide RNA (sgRNA) sequence was extracted, and an on-target score was computed using the Azimuth 2.0 algorithm^15^. In our approach, we employed a more stringent selection criterion using a score cutoff of 0.5 for highly efficient sgRNAs, in contrast to the recommended 0.2 (Fig. 1d, Step 6). Next, we screened potential off-target sites for each sgRNA using the Cas-OFFinder algorithm^16^. For sgRNAs with possible off-target regions, an off-target score was calculated using the Cutting Frequency Determination (CFD) method^15^. sgRNAs without off-target sites or with low off-target scores (< 0.175) were collected based on previously established cutoff^17^ (Fig. 1d, Step 7). Finally, we generated a comprehensive list of passenger SNVs suitable for CRISPR/Cas9-mediated cancer therapy and their corresponding sgRNAs (Supplementary Data 2). The selected passenger SNVs exhibited allele frequencies comparable to those of the oncogene hotspot mutations, which were significantly higher than those before selection (Fig. 1e). When we applied our approach to the TCGA dataset, we found that, on average, 44% in each cancer type (with a maximum of 72.1% for head and neck squamous cell carcinoma, HNSC) had at least one applicable passenger SNV for CRISPR/Cas9-mediated gene therapy. However, there was limited applicability in certain cancers, such as glioblastoma multiforme (GBM) and kidney renal clear cell carcinoma (KIRC), potentially due to the paucity of early-stage passenger mutations that met the AF cutoff (Fig. 1f). In summary, among the 6893 patients analyzed in TCGA dataset, 3097 (44.9%) had at least one eligible passenger SNV, which was approximately three times higher than the prevalence of oncogene hotspot mutations (14.4%) (Fig. 1g).

### Experimental validation of the therapeutic strategy in human colorectal cancer cell lines

**Figure 2:**
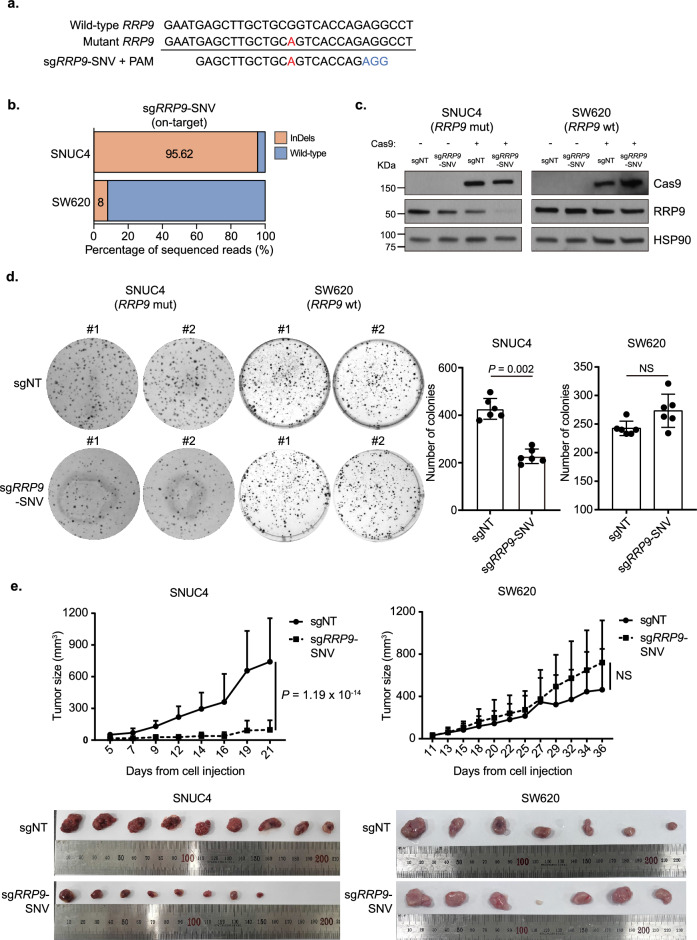
Experimental validation of the therapeutic strategy with essential gene *RRP9* in human colorectal cancer cell lines. (**a**) Genomic DNA sequences of wild-type *RRP9* and *RRP9* of the SNUC4 cell line, and sgRNA with the protospacer adjacent motif (PAM) sequence targeting passenger single-nucleotide variant (SNV) of SNUC4 *RRP9*. The SNV is represented in red and the PAM sequence in blue. (**b**) Frequency of insertions and deletions (InDels) and wild-type reads from *RRP9*-targeted next-generation sequencing in sg*RRP9*-SNV-transduced SNUC4 and SW620 cells. (**c**) The effect of sgRNA targeting the *RRP9* SNV of SNUC4 on RRP9 protein levels was assessed via immunoblotting whole-cell lysates from SNUC4 and SW620 cell lines. sgNT, non-targeting sgRNA. (**d**) Effect of sgRNA targeting *RRP9* SNV of SNUC4 on colony formation of SNUC4 and SW620 cell lines. (Left) Representative images from the colony formation assay and (right) quantification of crystal violet-stained colonies. The statistical significance of the difference in colony numbers between groups was assessed using a two-sided Wilcoxon rank-sum test. Data are presented as mean ± standard deviation (s.d.). NS, not significant. (**e**) Effect of sgRNA targeting the *RRP9* SNV of SNUC4 on (left) SNUC4 and (right) SW620 tumor growth. The statistical significance of the difference in tumor growth between groups was determined using two-way ANOVA. Data are presented as mean ± standard deviation (s.d.). Images show the dissected tumors after being sacrificed. NS, not significant.

To validate our strategy, we implemented our approach in human colorectal cancer cell lines and generated candidate passenger SNVs and their corresponding sgRNAs (Supplementary Data 3). We further filtered the SNVs by selecting those that were also identified as somatic mutations in at least one TCGA patient with colorectal cancer. From these candidates, we identified a distinctive passenger SNV (chromosome 3, position 51,970,353, missense variant, reference allele: G, variant allele: A) within the essential ribosomal gene *RRP9* of the SNUC4 cell line. This SNV was exclusive to the SNUC4 cell line and was not present in any other colorectal cancer cell line or normal tissue in TCGA cohort (Fig. 2a). Next, we sought to selectively cleave the mutant *RRP9* allele while sparing the wild-type *RRP9* allele to induce mutant allele-specific cell death. We used the SW620 colorectal cancer cell line, which carries wild-type *RRP9*, as a control. SNUC4 and SW620 cells, stably expressing the Cas9 protein, were transduced with a lentivirus containing an sgRNA targeting the SNUC4 *RRP9* SNV (*RRP9* g.51970353G > A; sg*RRP9*-SNV). As expected, next-generation sequencing (NGS) and the T7E1 assay confirmed highly efficient InDels in SNUC4 cells, but not in SW620 cells (Fig. 2b, Supplementary Fig. 1a,b). Indeed, the RRP9 protein was depleted only in SNUC4 cells but not in SW620 cells (Fig. 2c). No off-target DNA cleavage was observed in SNUC4 cells due to sg*RRP9*-SNV (Supplementary Fig. 1c). In addition, the loss of RRP9 resulted in defective colony formation and cell growth exclusively in SNUC4 cells, whereas SW620 cells remained unaffected (Fig. 2d, Supplementary Fig. 1d). The cell death induced by the loss of RRP9 in sg*RRP9*-SNV transduced SNUC4 cell lines was rescued by exogenous expression of wild-type *RRP9* (Supplementary Fig. 1e,f). To assess whether this phenomenon could be recapitulated in vivo, we subcutaneously injected mice with SNUC4 and SW620 cells stably expressing Cas9, transduced with a lentivirus carrying either a non-targeting sgRNA or an sgRNA targeting the SNUC4 *RRP9* g.51970353G > A, into their flanks. Intriguingly, tumor growth was significantly suppressed only in SNUC4 tumors, whereas no effect was observed in SW620 tumors (Fig. 2e).

To validate our approach targeting haploinsufficient gene-associated passenger SNVs, we identified a distinct heterozygous passenger SNV (chromosome 17, position 2,203,354, synonymous variant, reference allele: G, variant allele: A, AF: 0.58) within the haploinsufficient nonsense-mediated mRNA decay gene *SMG6* in the NCIH498 cell line. This SNV was exclusive to NCIH498 and absent in other colorectal cancer cell lines or normal tissues within the TCGA cohort (Supplementary Fig. 2a). We sought to selectively induce mutant allele-specific cell death by cleaving the mutant *SMG6* allele while preserving the wild-type *SMG6* allele. Using the SW620 cell line as a wild-type control, both NCIH498 and SW620 cell lines, stably expressing the Cas9 protein, were transduced with a lentivirus containing sgRNA targeting the NCIH498 *SMG6* SNV (*SMG6* g.2203354G > A; sg*SMG6*-SNV). The T7E1 assay confirmed exclusive non-homologous end joining in NCIH498 cells, not observed in SW620 cells (Supplementary Fig. 2b,c). The loss of *SMG6* resulted in decreased cell viability exclusively in NCIH498 cells, with no impact on SW620 cells (Supplementary Fig. 2d). Therefore, these findings substantiate the efficacy of our strategy in targeting passenger SNVs within a haploinsufficient gene.

## Discussion

Current approaches to anti-cancer CRISPR therapy predominantly focus on targeting oncogenic hotspot mutations or multiple InDels, limiting their applicability to a small subset of patients. However, it is evident that most tumor mutations are passenger mutations, and approximately 3000 human genes are haploinsufficient or essential. Therefore, our strategy, which targets passenger mutations in haploinsufficient or essential genes, specifically inducing the death of cancer cells carrying these mutations, can significantly overcome the limitations of existing strategies. We anticipate that, on average, 44.8% of patients with solid cancers could be targeted using our approach. This represents a substantial improvement over the current strategy, which targets oncogenic hotspot mutations and is applicable to only 14.4% of solid cancer patients. The limitation of our approach lies in the inefficient delivery of CRISPR components in in vivo conditions and its potential toxicity to normal cells due to off-target effects. However, as CRISPR technology continues to progress, we anticipate the development of highly efficient delivery methods and precise DNA editing techniques with enhanced single-nucleotide resolution, thereby minimizing off-target cleavage. Indeed, the safety and efficacy of CRISPR therapy have been demonstrated for treating genetic diseases caused by a single gene^18^. Furthermore, recent clinical trials employing CRISPR/Cas9 for the treatment of various illnesses, including cancer, liver diseases, sickle cell disease, and cardiovascular diseases, have been conducted^18^. These trials anticipate successful translation of the technology to the clinical realm.

## Methods

### TCGA data acquisition and pre-processing

TCGA SNV data for 16 cancer types (BLCA, BRCA, COAD, GBM, HNSC, KIRC, KIRP, LIHC, LUAD, LUSC, OV, PAAD, PRAD, READ, STAD, and UCEC) were downloaded from the GDC data portal (https://portal.gdc.cancer.gov/, DR-7.0). The mutation files were initially collected as VarScan2 processed protected mutation annotation format (MAF) files. To eliminate low-quality and potential germline variants, we further processed the files according to the guidelines provided by the GDC portal (https://docs.gdc.cancer.gov/Data/File_Formats/MAF_Format/) to generate high-confidence somatic mutation files. For gene expression analysis, we obtained fragments per kilobase of exon per million mapped fragments (FPKM) data using the TCGAbiolinks^19^ R package (version 2.26.0). The gene expression values were then normalized to log2(FPKM + 1).

### Essential and haploinsufficient gene and oncogene hotspot mutation annotation

The DepMap CRISPR/Cas9 screen dataset^20^ (https://depmap.org/portal/, DepMap Public 21Q2) was used to collect essential genes. Haploinsufficient genes were compiled from three sources: (1) Vinh T Dang et al.’s study^11^, (2) ClinGen^12^ (https://clinicalgenome.org, genes with haploinsufficiency scores of 2 or 3, downloaded on January 20, 2021), and (3) DECIPHER^13^ (https://deciphergenomics.org, genes located in the top 5% probability of haploinsufficiency scores, version 3). Oncogenes were obtained from the COSMIC Cancer Gene Census^9^ (https://cancer.sanger.ac.uk/census, v94) data by applying the filter “Somatic = yes” and including genes with the role of “oncogene” in cancer. Hotspot mutations were annotated using data from the Cancer Hotspots portal^3^ (https://www.cancerhotspots.org, Hotspot Results V2).

### Targetable SNV selection and sgRNA sequence generation

To generate targetable SNVs and the corresponding sgRNA sequences from a given SNV list of a sample, we followed the following steps: First, we identified the SNVs located within essential or haploinsufficient genes. If an SNV was encoded by an essential gene, only homozygous SNVs were further analyzed. Next, we calculated the allele frequency (AF) threshold ${AF}_{cut}$ using the following equation:

1 $${AF}_{cut}={AF}_{M}+MAD(hetAF)$$

where ${AF}_{M}$ is the median of AFs of SNVs from the sample, and $MAD(hetAF)$ is the median absolute deviation (MAD) of AFs of heterozygous SNVs from the patient or sample. SNVs with AF below the sample’s ${AF}_{cut}$ were filtered out. We then considered the expression of the gene in which an SNV was located and retained SNVs where the gene expression (log_2_(FPKM + 1)) was greater than 1.

To identify SNVs that generate a novel and specific targetable site for the CRISPR/Cas9 approach, we searched for a PAM sequence (NGG, where N represents any nucleotide) within a 12-base pair region around the SNV or checked if the SNV itself created a new PAM sequence. For the satisfying SNVs, a 20-nucleotide sgRNA sequence was obtained.

To obtain sgRNAs with precise knockout efficiency and low potential off-target effects, we calculated the on- and off-target scores and applied strict cutoffs as follows: First, on-target scores were calculated using the Azimuth 2.0^15^ method implemented in the crisprScore^21^ R package (version 1.2.0). sgRNAs with on-target scores greater than 0.5 were examined for possible off-target sites using CasOFFinder^16^ (offline version 2.4). The UCSC human reference genome assembly (GRCh38) was used as a reference, and off-target sites with a maximum of three mismatches were searched. If an sgRNA was found to have off-target sites, the off-target score was calculated using the CFD^15^ method, which was also implemented in the crisprScore^21^ R package. If off-target sites with scores > 0.175 were present, the sgRNA was filtered out to mitigate potential off-target risks. Finally, the SNVs were reported along with their corresponding sgRNAs, on-target scores, and off-target scores.

### Cell lines and culture

All cells were maintained at 37 °C in a 5% CO_2_ atmosphere. Human embryonic kidney 293 T (HEK293T) cells were purchased from ATCC. HEK293T cells were cultured in Dulbecco’s modified Eagle’s medium (DMEM) (Gibco, USA) supplemented with 10% fetal bovine serum (FBS) (Gibco) and 1% penicillin–streptomycin (Invitrogen, USA). Human colorectal cancer cell lines (SNUC4, SW620, and NCIH498) were also purchased from the Korean Cell Line Bank and cultured in RPMI-1640 medium (Gibco) supplemented with 10% FBS and 1% penicillin–streptomycin.

### Lentiviral transduction

The lentiviral plasmids lentiCas9-Blast and lentiGuide-puro were purchased from Addgene USA (#52,962, #52,963). The sgRNA sequences were cloned following the lentiCRISPR v2 cloning protocol^22,23^. For transfection, 7.5 × 10^5^ HEK293T cells were seeded in 60-mm plates one day before transfection. Transfection was performed using Opti-MEM™ I Reduced Serum Medium (Gibco) with 1 μg of lentiviral plasmid, 0.25 μg of pMD2.G (#12,259; Addgene), 0.75 μg of psPAX2 (#12,260; Addgene), and 6 μL of FuGENE^®^ (Promega, USA). The medium was changed after 16 h of incubation at 37 °C under 5% CO_2_. Viral supernatants were collected 48 and 72 h after transfection, filtered through a 0.45-μm membrane (Corning, USA), and stored at -80 °C. Cells were transduced with lentivirus encoding lentiCas9-Blast to establish stable Cas9-expressing cells, followed by selection with blasticidin (10 μg/mL) (Invitrogen) for seven days.

### Colony formation assay

Stable Cas9-expressing SNUC4 and SW620 cells were transduced with a lentivirus encoding either control sgRNA (non-targeting sgRNA, GCGAGGTATTCGGCTCCGCG) or sgRNA targeting the *RRP9* SNV of SNUC4 (sg*RRP9*-SNV). After selection with puromycin (SNUC4: 10 μg/mL, SW620: 2 μg/mL, Invitrogen) for 72 h, 1 × 10^3^ cells/well were seeded into six-well plates. The medium was replaced every 72 h. After 14 days, the medium was removed, and the cells were stained with 0.05% crystal violet solution in a 6% glutaraldehyde solution for 30 min. The crystal violet solution was then removed, and the cells were washed with H_2_O and allowed to dry. Colonies comprising more than 50 cells were counted using the ImageJ software^24^.

### Cell growth assay

Parental or stable Cas9-expressing SNUC4 and SW620 cells were transduced with a lentivirus encoding either control sgRNA (non-targeting sgRNA, GCGAGGTATTCGGCTCCGCG) or sg*RRP9*-SNV. After selection with puromycin (SNUC4: 10 μg/mL, SW620: 2 μg/mL) for 72 h, 1 × 10^5^ cells/well were seeded into six-well plates. After 3 days, cells were trypsinized, stained with trypan blue (Bio-Rad, USA), and counted. All harvested cells were seeded onto 60-mm plates. After 3 days of incubation, cells were trypsinized and counted with trypan blue again. The subculture was repeated once more using 100-mm plates. Growth curves were generated using cell counts obtained during the subculture.

### *RRP9* rescue assay

Total RNA was extracted from SW620 cell line using the RNeasy Plus Mini Kit (QIAGEN, Germany) following the manufacturer’s instructions. cDNA was synthesized with PrimeScript™ RT Master Mix (Takara Korea Biomedical Inc, Korea), and full-length *RRP9* cDNA was PCR amplified with CloneAmp HiFi PCR Premix (Takara Korea Biomedical Inc). The PCR-amplified *RRP9* wild-type cDNA was cloned into pcDNA3 Flag HA (#10,792, Addgene) using In-Fusion HD^®^ Cloning Kit (Takara Korea Biomedical Inc). *RRP9* sequence was confirmed by Sanger-sequencing.

Stable Cas9-expressing SNUC4 cells were transduced with lentivirus encoding either control sgRNA (non-targeting sgRNA, GCGAGGTATTCGGCTCCGCG) or sg*RRP9*-SNV. After selection with puromycin (10 μg/mL) for 72 h, 3 × 10^3^ cells/well were seeded into 96-well plates. After a 24 h incubation, 2 μg of empty or *RRP9* plasmids were transfected with FuGene HD (Promega) according to the manufacturer’s protocol. Cell viability was assessed after 4 days using Cell Titer Glo (Promega), and relative luminescence units (RLU) were measured using an EnVision plate reader (Perkin-Elmer, USA).

### Cell viability assay

Stable Cas9-expressing NCIH498 and SW620 cells were transduced with a lentivirus encoding either control sgRNA (non-targeting sgRNA, GCGAGGTATTCGGCTCCGCG) or sgRNA targeting the *SMG6* SNV of NCIH498 (sg*SMG6*-SNV). After selection with puromycin (NCIH498: 10 μg/mL, SW620: 2 μg/mL) for 72 h, 3 × 10^3^ cells/well were seeded into 96-well plates. After 6 days, cell viability was determined with Cell Titer Glo according to the manufacturer’s protocol, and RLU were measured using an EnVision plate reader.

### Western blotting

Cells and tissues were harvested, washed with phosphate-buffered saline (PBS), and lysed on ice for 15 min in a radioimmunoprecipitation assay buffer (R0278; Sigma, USA) supplemented with a protease and phosphatase inhibitor cocktail (GenDEPOT, USA). Cell lysates were centrifuged at 4 °C for 10 min at 15,000 rpm. Protein concentrations were determined using Bradford assay (Bio-Rad). Equal amounts of total protein were separated via sodium dodecyl sulfate gel electrophoresis and transferred to polyvinylidene difluoride membranes (Bio-Rad). The membranes were blocked with 5% skim milk for 1 h at 22 °C and then incubated overnight at 4 °C with a primary antibody against the target protein in a buffer containing 0.1% Tween 20. Subsequently, the membranes were washed with Tween-PBS buffer three times for 10 min each and incubated with a secondary antibody (anti-rabbit IgG or anti-mouse IgG) diluted in a blocking buffer containing 0.1% Tween 20 for 1 h at 22 °C. The membranes were then washed with Tween-PBS three times for 10 min each. The immunoreactive bands were visualized using Pierce enhanced chemiluminescence western blotting substrate (32,106; Thermo Fisher Scientific, USA). Mouse monoclonal anti-Cas9 (#14,697; Cell Signaling Technology, USA), rabbit polyclonal anti-RRP9 (#ab168845, Abcam, UK), rabbit polyclonal anti-FLAG (DYKDDDDK) (#2368; Cell Signaling Technology) and rabbit monoclonal anti-heat shock protein 90 (HSP90) (#4877, Cell Signaling Technology) and were used at a 1:1000 dilution. Anti-rabbit IgG (#111-035-144; Jackson ImmunoResearch, USA) was used at a 1:5000 dilution except for anti-FLAG which was used at a 1:10,000 dilution. Anti-mouse IgG (#115-035-146, Jackson ImmunoResearch) was used at a 1:10,000 dilution.

### InDel quantification via targeted next generation sequencing

Genomic DNA was extracted using the QIAamp DNA Mini Kit (QIAGEN) following the manufacturer’s instructions. Libraries were prepared with a two-step PCR reaction, in which the first step uses target-specific primers, and the second step utilizes primers containing unique barcodes and Illumina sequencing adaptor sequences. The primers used here are listed in Supplementary Data 4. PCR reactions were performed with KAPA HiFi HotStart Ready Mix (Roche Molecular Systems, Inc. USA). For the first PCR step, 100 ng of genomic DNA was denatured at 95℃ for 5 min, followed by 30 cycles of (98 °C at 20 s, 61 °C for 15 s, and 72 °C for 15 s), and a final extension at 72 °C for 1 min. Primers with unique barcodes and Illumina sequencing adaptor sequences were added to the PCR product from step 1 for the second PCR reaction, where denaturation at 95 °C for 5 min was followed by 12 cycles of (98 °C at 20 s, 61 °C for 15 s, 72 °C for 15 s), and a final extension at 72 °C for 1 min. PCR products were verified with 2% agarose gel electrophoresis and extracted using the Zyomoclean Gel DNA Recovery Kit (Zymo Research, USA) according to the manufacturer’s instructions. The barcoded PCR products were pooled and subjected to paired-end sequencing (2 × 150 bp reads) on an Illumina NovaSeq-6000 instrument (Macrogen, Korea). InDel quantification was conducted using CRISPResso2^25^ with default parameters.

### T7 endonuclease 1 assay

Genomic DNA was extracted from colorectal cancer cell lines using the QIAamp DNA Mini Kit (QIAGEN) following the manufacturer’s instructions. Target regions were PCR-amplified with nTaq (Mg2 + plus) (Enzynomics, Korea) with the following primers: sg*RRP9-*SNV region (Forward: 5′-TCAAGGCCCTCGTTGATTCC-3′, Reverse: 5′-TTTTTGGGCTTTGTGGCTGC-3′), sg*SMG6*-SNV region (Forward: 5′-TCTGCATCGAAAGTGACACGA-3′, Reverse: 5′- CTATCAGCCTGGACGACGTTT-3′). PCR products were purified with PureLink Quick PCR Purification Kit (Invitrogen). 200 ng of purified PCR product were denatured at 95 °C for 10 min, re-annealed at − 2 °C per second temperature ramp to 85 °C, followed by a − 1 °C per second ramp to 25 °C. 1 μl of T7E1 enzyme (Enzynomics) was added to the heterocomplexed PCR product and incubated at 37 °C for 15 min. Products were electrophoresed on a 2% agarose gel using TAE buffer. Band intensities were measured with ImageJ, and the estimated non-homologous end joining (NHEJ) event was calculated with the following formula:

2 $$NHEJ\left( \% \right) = 100 \times \left[ {1 - \left( {1 - fraction\; cleaved} \right)^{{\left( {\frac{1}{2}} \right)}} } \right]$$

where the fraction cleaved is $\frac{(Density\; of\; digested\; products)}{(Density\; of\; digested\; products\,+\,undigested\; parental\; band}$.

### Animal study

All animal procedures were approved by the Institutional Animal Care and Use Committee of Yonsei University, Seoul, Korea (2021-0106). All methods were performed in accordance with the relevant guidelines and regulations for the care and use of laboratory animals. Six-week-old female BALB/c-nu Slc mice were purchased from Orient Bio (Korea) and SLC Inc. (Japan). The mice were housed in individual ventilation cages equipped with a computerized environmental control system (Techniplast, Italy). The animal room temperature was maintained at 22 ± 2 °C with a relative humidity of 50 ± 10%. Before the experiments, the animals were acclimated for seven days under a 12-h light–dark cycle.

Stable Cas9-expressing SNUC4 cells were transduced with lentivirus encoding either the control sgRNA or sgRNA targeting the *RRP9* SNV in SNUC4 cells. After selection with 10 μg/mL puromycin for 72 h, 3 × 10^6^ cells were subcutaneously injected into the left (control sgRNA) or right (*RRP9* SNV of SNUC4 sgRNA) flanks of 10 mice. Similarly, stable Cas9-expressing SW620 cells were transduced with lentivirus encoding either the control sgRNA or sgRNA targeting the *RRP9* SNV in SNUC4 cells. After selection with 2 μg/mL puromycin for 72 h, 2 × 10^6^ cells were subcutaneously injected into the left (control sgRNA) and right (*RRP9* SNV of SNUC4 sgRNA) flanks of 10 mice. Among the mice, we excluded those with no observable tumor growth in the left flank (control sgRNA) from further analysis.

Tumor sizes were measured using a caliper, and the volume was calculated using the formula: 0.5 × length × width^2^. Mice were sacrificed when the largest tumor reached a volume of 1000 mm^3^. Each tumor was considered an experimental unit. The sample size was determined to be sufficient to identify statistically significant differences between groups.

### Whole-exome sequencing

Genomic DNA was extracted from colorectal cancer cell lines using the QIAamp DNA Mini Kit (QIAGEN) following the manufacturer’s instructions. Whole-exome capture was performed using the SureSelect Human All Exon V4 51 Mb Kit (Agilent Technologies, USA). The captured DNA was then sequenced on the HiSeq 2500 platform (Illumina, USA), generating a minimum of 98.9 million paired-end sequencing reads of 100 bp per sample.

The Burrows-Wheeler Alignment^26^ tool was used with the default parameters to align the paired-end reads to the UCSC human reference genome assembly (GRCh37/hg19). An average of 98.3% of the reads were successfully aligned to the human genome. Duplicate reads were removed using the Picard software package. The Genome Analysis Tool Kit (GATK) version 3.4–46 was used for read quality score recalibration and local realignment to identify short InDels using the HaplotypeCaller^27^ package. The variants were filtered using the GATK Best Practices quality control filters.

SNVs were identified using Mutect^28^, specifically the tumor-only option, with default parameters. Variants supported by at least five high-quality reads (Phred-scaled quality score > 30) and detected with at least 20% AF were selected for further analysis. The detected SNVs and InDels were annotated using various databases, including the single nucleotide polymorphism (SNP) database (dbSNP^29^, build 147), 1000 Genomes Project^30^ (Phase 3), Korean dbSNP (build 20,140,512), and somatic mutations in TCGA colon adenocarcinoma (COAD), using the Variant Effect Predictor software^31^ (version 87). ANNOVAR^32^ was used to annotate regions of known germline chromosomal segmental duplications and tandem repeats.

Several steps were performed to filter variants. Patients with germline polymorphisms, chromosomal segmental duplications, or tandem repeats were excluded. The variants were then filtered to include known somatic mutations observed in at least one sample from TCGA COAD dataset. Additionally, nonsynonymous mutations observed in genes belonging to the Cancer Gene Census, as reported in at least ten samples in the COSMIC^9^ database (version 87), were included in the analysis.

### RNA sequencing

Total RNA was extracted from colorectal cancer cell lines using the RNeasy Plus Mini Kit (QIAGEN) following the manufacturer’s instructions. The TruSeq RNA Sample Prep Kit v2 (Illumina) was used to generate mRNA-focused libraries. Libraries were sequenced on the HiSeq 2500 platform, generating at least 40 million paired-end reads of 100 bp per sample.

The TopHat-Cufflinks^33^ pipeline was employed to align the reads to the reference genome and calculate normalized gene expression values in FPKM. TopHat was used to align and map the reads to the reference genome. The resulting alignments were then processed using Cufflinks, which estimates transcript abundance and calculates FPKM values, providing a measure of gene expression levels that takes the length of exons and the total number of mapped reads into account.

### Software

R (ver. 4.2.1) (R Foundation, Austria) and the ImageJ software were used to analyze the data.

### Statistical analysis

The figures were generated using the R software, and statistical analyses were performed using RStudio software (version 2022.07.2 + 576). Specific statistical tests are identified in the figure legends for each experiment.

### Study reporting

The study design, animal use and all experimental methods were conducted and reported in accordance with ARRIVE guidelines (https://arriveguidelines.org).

### Supplementary Information

Supplementary Information 1.

Supplementary Information 2.

Supplementary Information 3.

Supplementary Information 4.

Supplementary Figure 1.

Supplementary Figure 2.

Supplementary Figure 3.

Supplementary Figure 4.

## Data Availability

TCGA data are available at the GDC data portal (https://portal.gdc.cancer.gov/). Whole exome sequencing and RNA-sequencing data are available at the Sequence Read Archive (SRP454320).

## Code availability

The code for producing cancer gene therapy-actionable passenger SNVs using CRISPR/Cas9 is available for download at https://github.com/hk-lab-software/crisprSNV2022/.
