# Supplementary material for: CRISPR/Cas9 targeting of passenger single nucleotide variants in haploinsufficient or essential genes expands cancer therapy prospects
Source: Sci Rep. 2024 Mar 28;14:7436. doi: 10.1038/s41598-024-58094-8 (PMC10978915; doi:10.1038/s41598-024-58094-8)
Supplement: Supplementary file 5 — Supplementary Figure 1. [file 41598_2024_58094_MOESM5_ESM.pdf]

a.

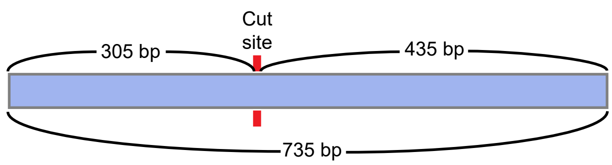

b.

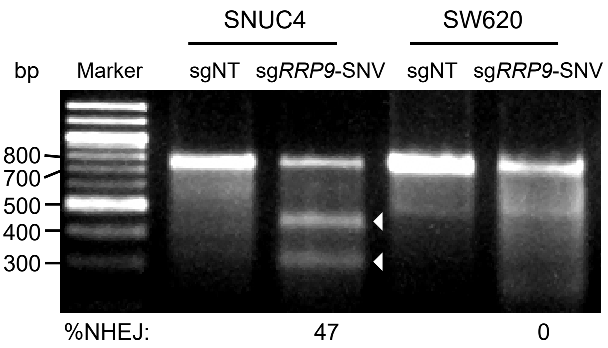

c.

sgRRP9-SNV	Off-target (OT)	Chromosome	Position	DNA	Msimatches	CFD scores
GAGCTTGCTGCAG TCACCAG	OT1	chr10	120762191	GAGCTGGCTGCAGTCTCTAGAGG	3	0
	OT2	chr12	116932226	GAGCTGGCTGCAGACAGCAGAGG		0.021
	OT3	chr2	238052896	GAGCTAGCTGCAGTCAGAAGAGG		0.027
	OT4	chr20	50817898	GAGCTCGCTGGAGTCACCTGAGG		0.122
	OT5	chr7	47083041	GAGCTTGTTGGAGACACCAGAGG		0.135
	OT6	chr10	90524986	GAGTTTTCTGCTGTCACCAGAGG		0.152

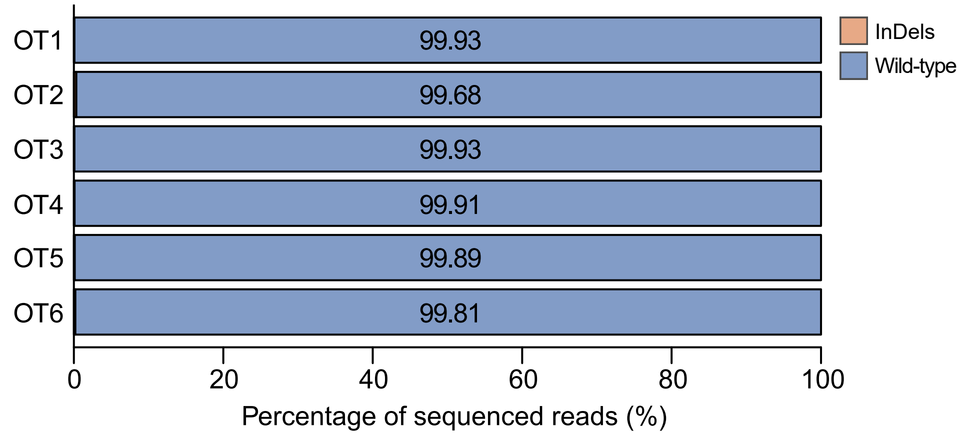

d.

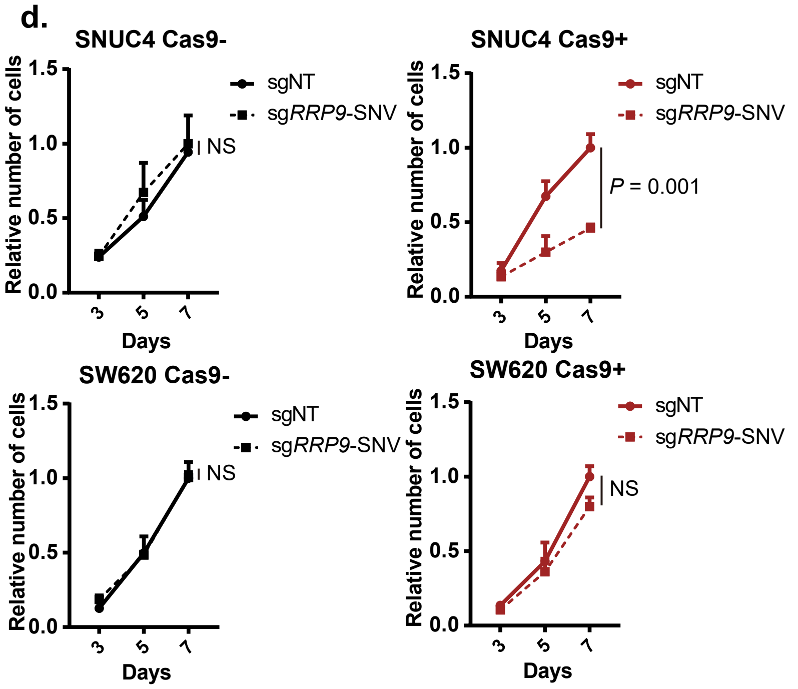

e.

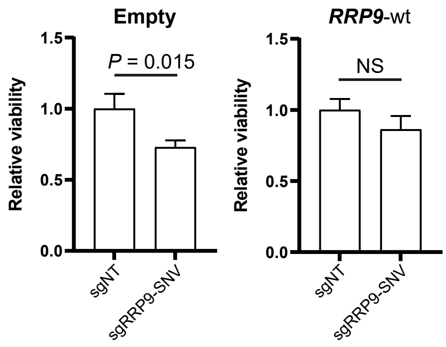

f.

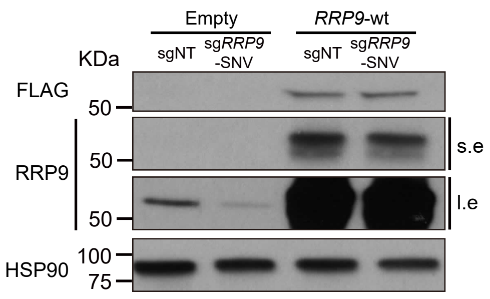

Supplementary Figure 1. Experimental validation of the therapeutic strategy with essential gene *RRP9* in human colorectal cancer cell lines, related to Figure 2. (**a**) Schematic of PCR amplicon design for T7E1 assay. (**b**) Non-homologous end joining (NHEJ) frequency with the T7E1 assay. Gel images of T7E1-treated PCR products amplified from the target site in control sgRNA-transduced or sg*RRP9*-SNV-transduced SNUC4 cells and SW620 cells. (**c**) Predicted off-target regions of sg*RRP9*-SNV (top) and frequency of InDels and wild-type reads from targeted next generation sequencing for the off-target regions in sg*RRP9*-SNV-transduced SNUC4 cells (bottom). (**d**) Effect of sgRNA targeting the *RRP9* SNV on cell growth of SNUC4 and SW620. Statistical significance of the difference in cell growth was determined using two-way ANOVA. Data are presented as mean ± standard deviation (s.d.). NS, not significant. (**e**) Overexpression of *RRP9* rescues SNUC4 cells against sg*RRP9*-SNV. Statistical significance of the difference in cell viability was determined using two-tailed Student t test. Data are presented as mean ± standard deviation (s.d.). (**f**) Immunoblotting of whole-cell lysates from SNUC4 cells transduced with sgNT or sg*RRP9*-SNV, overexpressing wild-type *RRP9* or empty vector. s.e, short exposure. l.e, long exposure.
