# Supplementary material for: CRISPR/Cas9 targeting of passenger single nucleotide variants in haploinsufficient or essential genes expands cancer therapy prospects
Source: Sci Rep. 2024 Mar 28;14:7436. doi: 10.1038/s41598-024-58094-8 (PMC10978915; doi:10.1038/s41598-024-58094-8)
Supplement: Supplementary file 6 — Supplementary Figure 2. [file 41598_2024_58094_MOESM6_ESM.pdf]

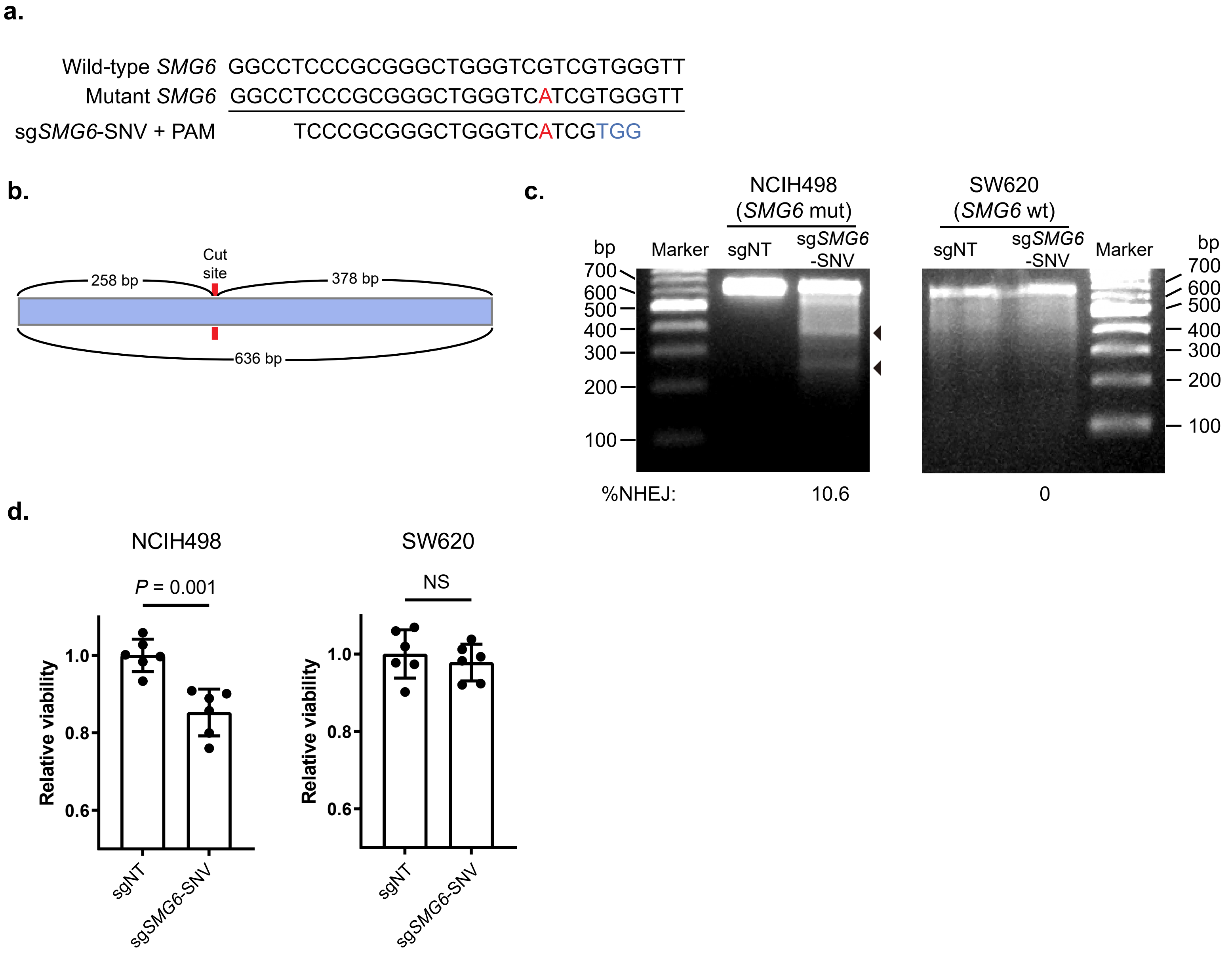

Supplementary Figure 2. Experimental validation of the therapeutic strategy with haploinsufficient gene *SMG6* in human colorectal cancer cell lines. (**a**) Genomic DNA sequences of wild-type *SMG6* and *SMG6* of the NCIH498 cell line, and single-guide RNA (sgRNA) with the protospacer adjacent motif (PAM) sequence targeting passenger single-nucleotide variant (SNV) of NCIH498 *SMG6*. The SNV is represented in red and the PAM sequence in blue. (**b**) Schematic of PCR amplicon design for T7E1 assay. (**c**) Non-homologous end joining (NHEJ) frequency with the T7E1 assay. Gel images of T7E1-treated PCR products amplified from the target site of sg*SMG6*-SNV in control sgRNA or sg*SMG6*-SNV transduced NCIH498 cells and SW620 cells. (**d**) Effect of sgRNA targeting the *SMG6* SNV on cell growth of NCIH498 and SW620. Statistical significance of the difference in cell growth was determined using two-tailed Student t test. Data are presented as mean ± standard deviation (s.d.). NS, not significant.
