# Supplementary material for: CRISPR/Cas9 targeting of passenger single nucleotide variants in haploinsufficient or essential genes expands cancer therapy prospects
Source: Sci Rep. 2024 Mar 28;14:7436. doi: 10.1038/s41598-024-58094-8 (PMC10978915; doi:10.1038/s41598-024-58094-8)

Supplementary Figure 3. Uncropped Western blot images of Figure 2b.

Cas9 (160 kDa)

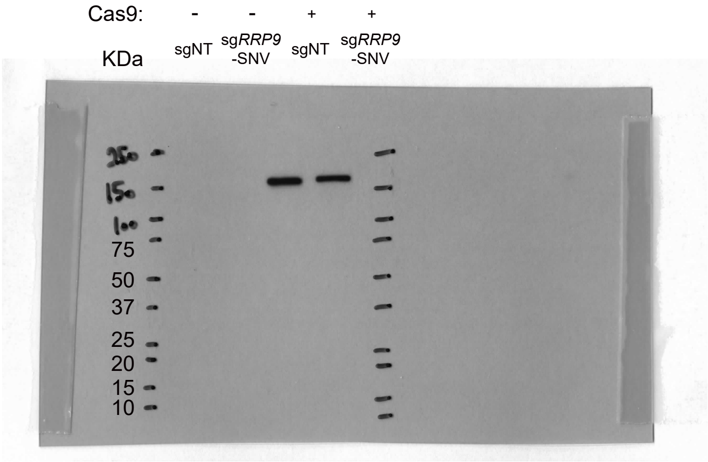

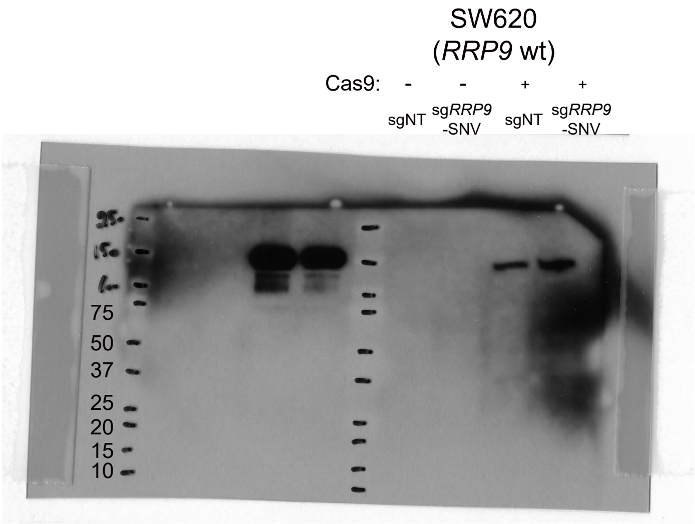

RRP9 (55 kDa)

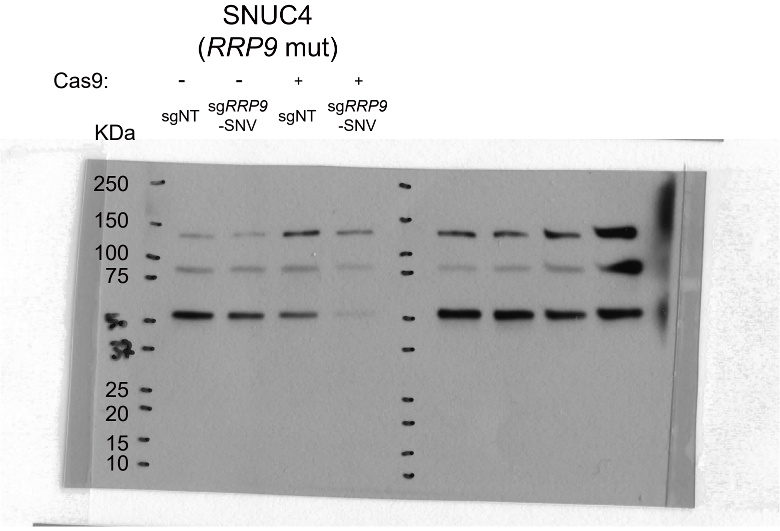

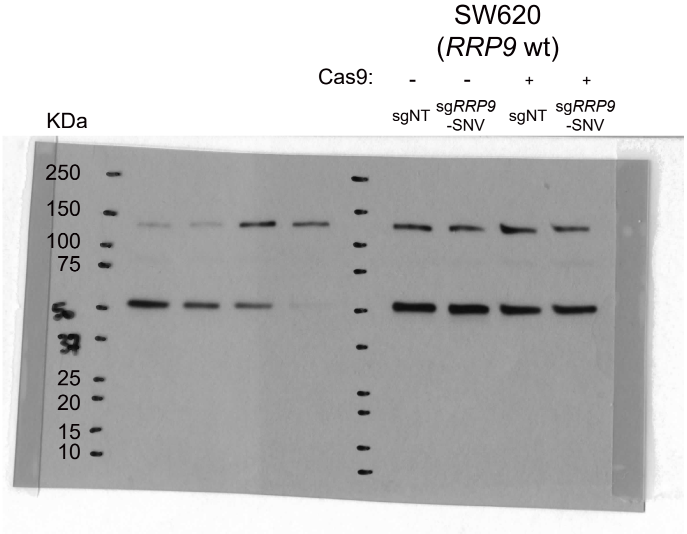

HSP90 (90 kDa)

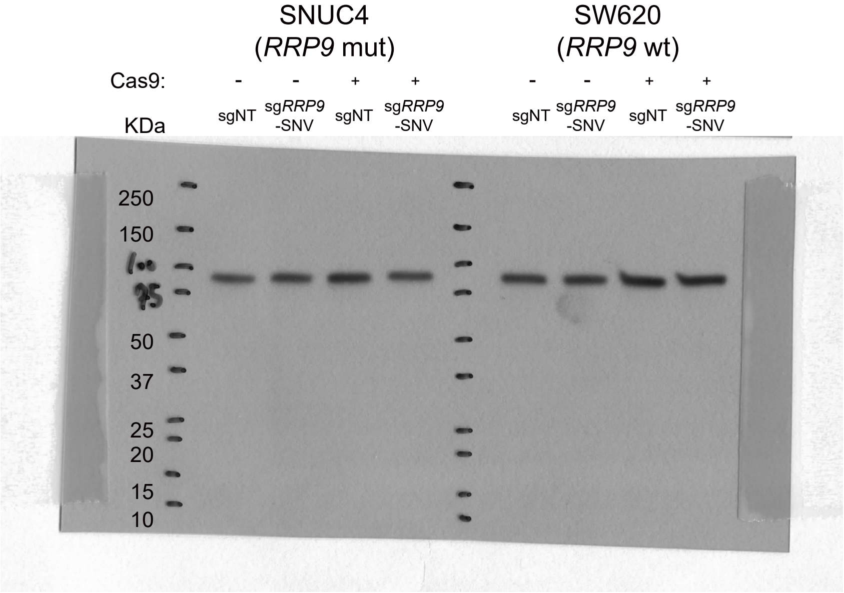

Supplement: Supplementary file 7 — Supplementary Figure 3. [file 41598_2024_58094_MOESM7_ESM.pdf]
