# Supplementary figures and images for: CRISPR/Cas9 targeting of passenger single nucleotide variants in haploinsufficient or essential genes expands cancer therapy prospects
Source: Sci Rep. 2024 Mar 28;14:7436. doi: 10.1038/s41598-024-58094-8 (PMC10978915; doi:10.1038/s41598-024-58094-8)

**Supplementary Figure 4.** Uncropped Western blot images of Supplementary Figure 1f.

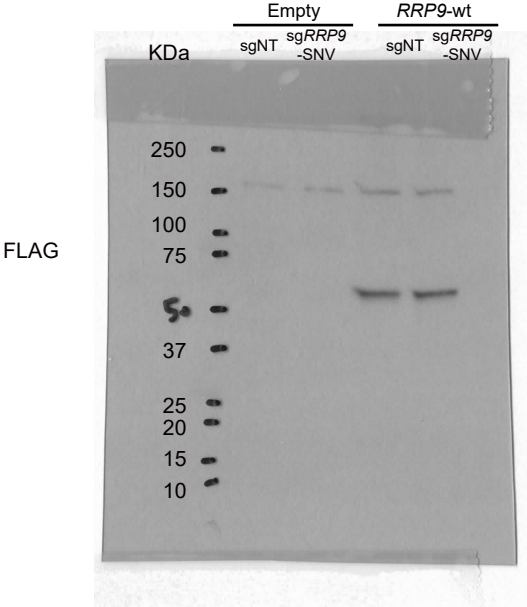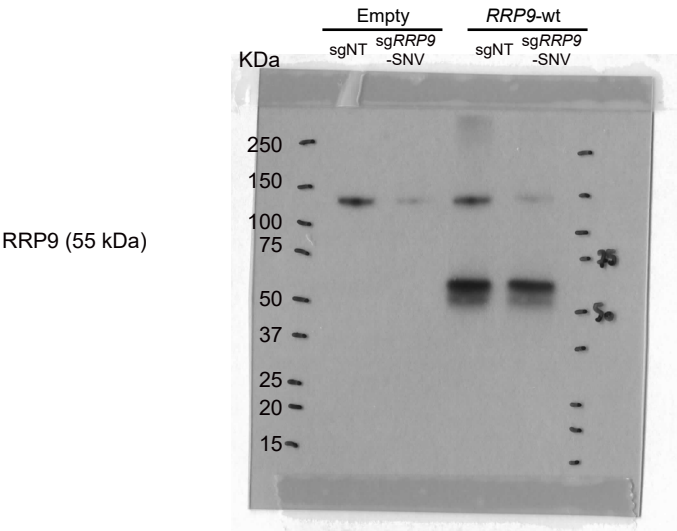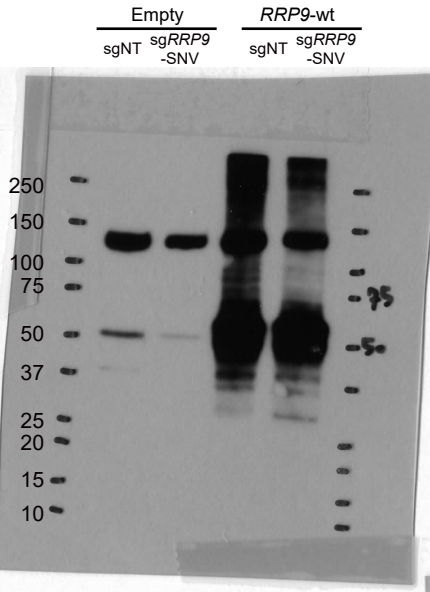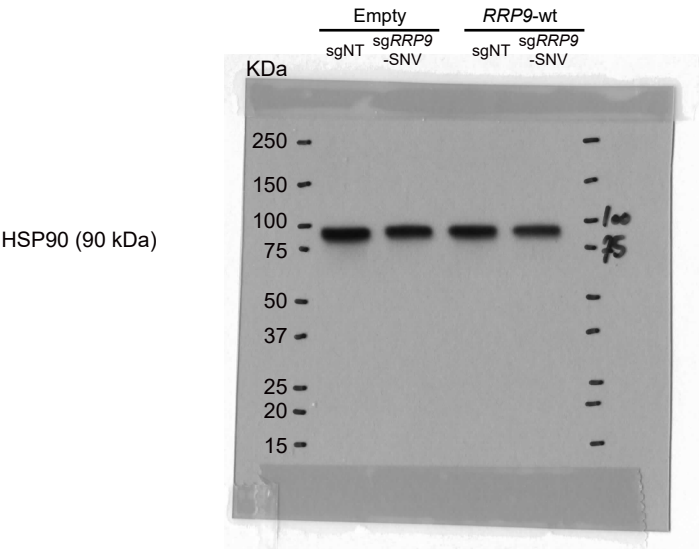

Supplement: Supplementary file 8 — Supplementary Figure 4. [file 41598_2024_58094_MOESM8_ESM.pdf]
